# How does social support affect public service motivation of healthcare workers in China: the mediating effect of job stress

**DOI:** 10.1186/s12889-021-11028-9

**Published:** 2021-06-05

**Authors:** Jianwei Deng, Jiahao Liu, Yuangeng Guo, Yongchuang Gao, Zhennan Wu, Tianan Yang

**Affiliations:** 1 School of Management and Economics, Beijing Institute of Technology, 5 Zhongguancun South Street, Beijing, 100081 China; 2 Sustainable Development Research Institute for Economy and Society of Beijing, 5 Zhongguancun South Street, Beijing, 100081 China

**Keywords:** Supervisor support, Coworker support, Job stress, Public service motivation

## Abstract

**Background:**

This study aimed to examine relations between social support, job stress, and public service motivation (PSM), also assessed how social support and job stress affect PSM in China based on the job demands-resources (JD-R) theory.

**Methods:**

The survey investigated a sample of 973 healthcare workers employed in public hospitals in Beijing, Xiamen, and Guangzhou in 2017 (including doctors, nurses, medical technicians, and administrators). Correlation analysis and Structural equation modeling (SEM) were used.

**Results:**

Challenge stress and hindrance stress were directly negatively associated with PSM. Supervisor support was significantly positively associated with PSM, and the path from coworker support to PSM was significant. Supervisor support was significantly negatively associated with hindrance stress, and coworker support was significantly negatively associated with challenge stress. Hindrance stress and challenge stress significantly mediated the relations between supervisor support and PSM, and between coworker support and PSM respectively. PSM might be raised by increasing supervisor support and coworker support and by limiting hindrance stress and challenge stress.

**Conclusion:**

Our study suggests that administrators of public hospitals should be mindful of the intense job stress of healthcare workers and undertake interventions targeting challenge stress and hindrance stress. Also, public hospital administrators should encourage and assist supervisors in their leadership functions. Besides, administrators of public hospitals should emphasize coworker support and good employee relationships.

**Supplementary Information:**

The online version contains supplementary material available at 10.1186/s12889-021-11028-9.

## Background

In order to set apart the motivational bases of public service from the traditional rational motives of other sectors [1], Perry and Wise developed the concept of public service motivation (PSM) to understand behavior and staff management in public organizations, e.g., public hospitals [2], which has generated a lively research discourse and considerable interest in recent decades [3, 4]. PSM has been defined as “the idea of commitment to the public service, pursuit of the public interest, and the desire to perform work that is worthwhile to society” [5]. Witteloostuijn et al. described how research on PSM has involved two main perspectives. The first perspective focused on the PSM itself, including the definition, composition and measurement of PSM [6]. The second perspective was concerned with the impact of PSM at the individual and organizational level, such as volunteering and person-organization fit [7, 8]. Compared to existing research treating PSM as an independent variable, to our best knowledge, few studies have examined PSM as a dependent variable or have investigated the antecedents of PSM, although some investigations have been primarily conducted (e.g., individuals’ demographic information and job satisfaction) [6, 9].

Recent studies have investigated these limitations in the theoretical and practical treatment of PSM [2, 10]. Sex, education level, religious activities, volunteer work, family socialization, leadership style, hierarchical authority, and red tape are widely accepted antecedents of PSM [1, 11]. However, evidence and mediators regarding these antecedents are limited. Social support has a positive effect on individual motivation [12], and therefore affect PSM directly or through other variables, e.g., job stress, a common mediator in studies of social support [13]. Given the level of experienced stress was associated with specific stressors, job stress was divided into challenge stress (e.g., individuals felt high level of job overload, responsibility, and time stress) and hindrance stress (e.g., individuals suffered red tape, unclear tasks, and worry about job security) [14, 15], which would have positive or negative effects on their job satisfaction or voluntary turnover [14]. Therefore, it’s necessary and interesting to investigate how social support and job stress affect PSM.

Social support can affect PSM. Social support usually occurs when a person we can rely on shows that they care, value, and love us [16], which can be divided into supervisor/coworker support and non-work support (e.g., family support, friend support, and partner support) [17]. Supervisor support and coworker support may be the most important social support factors affecting workers’ PSM. With sufficient supervisor support, workers feel they are important members of the organization and realize that their goals fit organizational goals [18]. They will thus be more involved in their work and demonstrate more altruistic behavior, dedication, and organizational citizenship behavior—the concrete manifestations of PSM. With support from coworkers, employees can work better and provide better public service. More importantly, such employees are more likely to be happy with their work, which strengthens their PSM. These could satisfy the psychological needs of employees and increase the complementary fit between the individual and organization [19]. Therefore, we propose the following hypotheses for an empirical analysis.

Hypothesis 1: Supervisor support is positively related to PSM.

Hypothesis 2: Coworker support is positively related to PSM.

Job stress can be affected by social support. Job stress is generally regarded as a multidimensional concept, and the models proposed usually include various putative sources of job stress. A frequently used classification divides job stress into two factors: challenge stress and hindrance stress [14]. Challenge stress is defined as the job stress that people can overcome which is conducive to long - term career prospects (e.g., individuals felt high level of job overload, responsibility, and time stress), while hindrance stress is a kind of job stress that people cannot overcome which is harmful to long - term career prospects (e.g., individuals suffered red tape, unclear tasks, and worry about job security) [15]. In the workplace, supervisor support and coworker support are social support dimensions closed to job stress and contribute to supportive communication. Supervisor support may have both an informational and emotional effect on workers. Informational supervisor support could help reduce worker role conflict and organizational politics [20]. From an emotional perspective, supervisor support might help promote self-esteem and work security [21]. With the help and cooperation of coworkers, employees have a reduced job load and less time urgency and may thus better perform their job responsibilities. In turn, they likely obtain more personal fulfillment and less challenge to meet their job requirements. More importantly, workers are less likely to experience anxiety and can maintain a good state of mind by communicating with coworkers in the same work culture and environment [22]. This analysis suggests the following research hypotheses.

Hypothesis 3: Supervisor support is negatively related to hindrance stress.

Hypothesis 4: Coworker support is negatively related to challenge stress.

Job stress can affect PSM and mediate the effect of social support on PSM. Considering different types of job stress, job stress would affect PSM through two principal mechanisms. On the one hand, challenge stress, e.g., job load, job responsibility, and time urgency increase the psychological burden and prevalence of mental disorders among staff [23], as well as the possibility of burnout and turnover intention [24]. However, if workers have sufficient professional skills, vocational relations, and coworker support, they may be able to adapt and overcome such challenge stress. Challenge stress would then be less harmful and might even encourage employees to increase their degree of job control and act positively to finish their work [25, 26]. In this situation, workers would feel that they are fit for the job and organization and able to perform the work, act in the public interest, and provide public service to others [27].

On the other hand, a sense of “effort-reward imbalance at work” also produces hindrance stress [28], and weakens workers’ PSM. For many employees, especially new workers, reciprocal modulation of the relationship between them and their supervisors is very important. While working hard, they also want to be recognized and rewarded by their supervisors and organization [18]. If their efforts elicit a positive response from supervisors, PSM is strengthened and they will work harder. However, if workers feel that supervisors are more focused on their social relations and professional background rather than on their job performance, or if workers are confused about their role responsibility and work process, they might experience frustration and a lack of job security [29]. More importantly, workers might come to regard their work as stagnant because of the lack of rewards and supervisor support. In these situations, workers felt huge hindrance stress, their enthusiasm and service motivation will be diminished. This analysis suggests the following research hypotheses.

Hypothesis 5: Challenge stress is positively related to PSM.

Hypothesis 6: Hindrance stress is negatively related to PSM.

Hypothesis 7: Challenge stress mediates the effect of coworker support on PSM.

Hypothesis 8: Hindrance stress mediates the effect of supervisor support on PSM.

Additionally, JD-R theory that is proposed to describe how job stress appears and affects employees’ psychological outcomes, provides further explains of the relations between social support, job stress and PSM [30]. This theory proposes that job characteristics can be divided into two opposed categories: job demands and job resources. Job demands refer to factors that require the individuals to make efforts or costs to complete the work, such as physical, psychological, and social abilities. Job resources are defined as factors that can (a) promote the achievement of employment objective, (b) reduce job demands and associated psychological and physical costs, and (c) promote personal prospect [31]. On the one hand, job demands lead to constant overtaxing and job stress, resulting in negative psychological and emotional consequences (e.g., exhaustion) and ultimately reduce PSM, as levels of stress accumulate over time will cause psychological withdrawal [32]. On the other hand, job resources directly and indirectly incentivize individuals. An important job resource—supervisor and coworker support—helps workers overcome job stress and enhances their PSM since this resource can alleviate the negative effect of job demands [30, 33, 34].

In China, public hospitals are an important part of the public sector, providing most healthcare services. The job characteristics, responsibilities and requirements of healthcare workers have led to increasing research interest in PSM and job stress among this group [35]. In addition, healthcare workers need sufficient social support in order to deal with complex working environment (e.g., overwork and violence) and provide good healthcare service. Social support, job stress, and PSM may therefore be a crucial and topic concern.

**Fig. 1:**
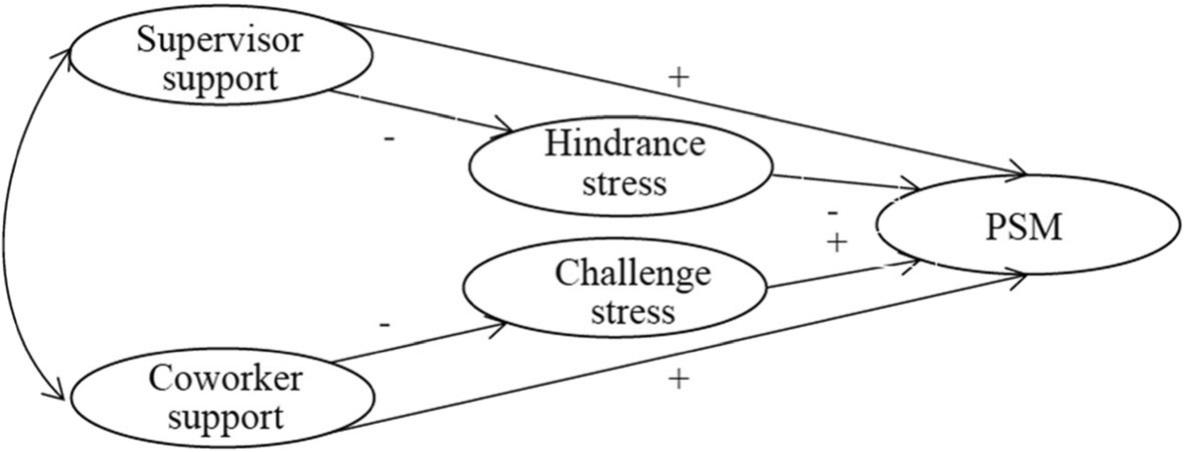
Proposed Model of How Supervisor Support and Coworker Support Influence Challenge Stress, Hindrance Stress and Public Service Motivation (PSM)

Most of the previous studies did not explore the antecedents of PSM, although some investigations have been primarily conducted [6, 9]. They did not consider the influence of social support and the mediating role of job stress. Thus, in this study we examined the mediating effects of job stress on the association between social support and PSM (Fig. 1), with a sample of Chinese healthcare workers from the area of knowledge part of a hospital context (e.g., doctors, nurses, medical technicians, and administrators) [36].

## Methods

### Participants and setting

We conducted a cross-sectional analysis of data from healthcare workers employed in public hospitals in Beijing, Xiamen, and Guangzhou in 2017 (including doctors, nurses, medical technicians, and administrators) to investigate the relationship between social support, job stress and PSM. To comply with the requirements of the research ethics committee, we first contacted the leaders of the target hospitals to obtain the approval. For hospitals that agreed to participate, we work with the relevant department, delivered the paper-based questionnaires to participants, and obtained their verbal consents. Participants that were willing to participate will be required to return and complete questionnaires to us. In the whole process, we guaranteed that all the questionnaires will be filled in an anonymous way, and the contents of the survey were used only for scientific research to improve their life and work quality, and absolute confidentiality. To ensure data integrity and objectivity, participants were selected by using random sampling according to employee number, age and job title. We finally included 5 to 10% of healthcare workers in each target hospital, including doctors, nurses, medical technicians, and administrators.

The survey assessed individual characteristics, supervisor support, coworker support, job stress, and PSM. To test convergent and divergent validity in our survey, we used similar and opposite questions in the questionnaire to examine whether our respondents provide the expected answers. The participants were finally excluded if their answers were not consistent. Finally, we included 93.8% of the total participants after excluding the effects of social desirability (973 valid participants including 315 doctors, 362 nurses, 108 administrators, 112 medical technicians).

### Measures

PSM was measured with the reliable five-item scale adapted from the original 40 items outlined by Perry [37]. This scale has been tested and used extensively in previous research [38–40]. The scale was shown to have high reliability in our research (α = 0.93). The participants needed to respond the items like “Meaningful public service is very important to me” and “I am often reminded by daily events about how dependent we are on one another” (see Additional file 1) and rate their PSM on a scale from 1 (strongly disagree) to 5 (strongly agree). The higher values they obtained, the greater PSM they owned.

Job stress scale was self- reported and designed with reference to the challenge- and hindrance-related stress scale (C-HSS) [14]. We measured challenge stress using 6 items and hindrance stress using 5 items. The participants needed to respond the items like “The number of projects and/or assignments I have” and “The lack of job security I have” (see Additional file 1) with a five-point Likert scale (1 = no stress; 5 = great stress). Higher values illustrate greater job stress. The C-HSS scale has been adopted in other research [40] and shown to have high reliability (α = 0.85–0.94) in the present study.

Social support from respondents’ supervisors and coworkers was measured with the four-item “supervisor support scale” (e.g.*, “My supervisor takes pride in my accomplishments at work”*) and the three-item “coworker support scale” (e.g. *“My coworkers help me in crisis situations at work”*) [41] (see Additional file 1). Each item was rated on a five-point scale (1 = strongly disagree; 5 = strongly agree). Higher values indicate greater support. The Cronbach α values for these scales were 0.92 for supervisor support and 0.90 for coworker support, in the HRS Psychosocial Working Group [34], and 0.92 and 0.83, respectively, for our study. Both these instruments have acceptable psychometric properties [42, 43].

All measures were originally constructed in English but have been translated and adopted in previous study in China. The job stress scale (α = 0.824–0.840) [44], PSM scale (α = 0.78) [39, 45], and social support scale (α = 0.914) [46] have showed a high reliability.

### Data analysis

We used two kinds of software for the statistical analyses. Descriptive analysis and correlation analysis were conducted in the SPSS 20.0, while path analysis in the AMOS 20.0. Structural equation modelling (SEM) analysis was used to deal with the common-source-bias in 2 steps [47, 48], and examine relationships among supervisor support, coworker support, challenge stress, hindrance stress, and PSM that are classified as direct or indirect [49]. The measurement equations of initial SEM are [50]:

$$ y={\Lambda}_Y\eta +\epsilon $$

$$ x={\Lambda}_X\xi +\delta $$

Where: *y* is a (p × 1) column vector of observed dependent variables. *x* is a (q × 1) column vector of observed independent variables. Λ_*Y*_ is a (p × m) regression coefficient matrix of *y* on *η*. Λ_*X*_ is a (q × n) regression coefficient matrix of *x* on *ξ*. *ϵ* is a (p × 1) column vector of errors of measurement in *y*. *δ* is a (q × 1) column vector of errors of measurement in *x*.

The linear structural equation of initial SEM is:

$$ {B}_{\eta }=\Gamma \xi +\zeta $$

Where: *B* is an (m × n) coefficient matrix. Γ is an (m × n) coefficient matrix. *η* is an (m × 1) column vector of constructs derived from the dependent variables (*y*). *ξ* is an (n × 1) column vector of constructs derived from the independent variables (*x*.). *ζ* is an (m × 1) column vector of the errors in the structural equations. m is the number of constructs (latent variables) developed from the observed dependent variables, and n is the number of constructs (latent variables) developed from the observed independent variable.

In SEM, five latent variables: PSM, challenge stress, hindrance stress, supervisor support and coworker support were first constructed with the items of the PSM scale, the C-HSS and social support scale. Before imputing these indicators into SEM, correlation analysis was used to determine the significance of correlations between PSM, challenge stress, hindrance stress, supervisor support and coworker support. All these indicators were examined to determine if the model fit the data well using confirmatory factor analysis.

Several recommendations have been made regarding adequate evaluation methods and sample sizes for non-normally distributed data when the normality test does not support the normality assumption for measured variables. Gold et al. insist that expectation-maximization implementation of maximum likelihood is much better than using the Asymptotically Distribution-Free Method on the model when the sample is over 500 [51]. As our study applied expectation-maximization in around 1000 participants, the method applied to evaluate model and sample size fulfilled both of these criteria.

Measures of local and global fit were checked when performing model testing. The local fit of the model was assessed on the basis of the following criteria: factor reliability values of 0.6 or more; indicator reliability value of 0.3 or more for each indicator of an underlying latent variable; *p* < .05 for all factor loadings and value of average proportions of indicator variance extracted 0.5 or more [52]. The criteria used to evaluate good global fit were chi-square minimal degrees of freedom (CMIN/DF < 5), a root mean square error of approximation (RMSEA) value of less than 0.05, the Goodness of Fit Index (GFI), Normed Fit Index (NFI), Comparative Fit Index (CFI) and Tucker-Lewis index (TLI) values of 0.90 or more [53]. The Sobel test was used to examine the significance of mediated effects [54].

To distinguish how the effects of different subgroups are varying, we conducted 5 subgroup analyses via the ages, sex, job title, post and seniority information. Age was categorized into three subgroups such as 41 years or older, 31–40 years and young 30 years or younger. Job title was divided into two categories as early career (trainee or entry-level worker) and mid−/late career (mid-level or senior worker). Sex was categorized as male and female. The post was classified into three subgroups, including doctors, nurses, and others (administrators and medical technicians). Seniority was classified as less than 5 years (employed for less than 5 years) and greater than 5 years (employed for longer than 5 years).

## Results

### Demographic characteristics

Our final sample consists of 5.35% (238 participants) of registered health professionals in the A hospital in Xiamen, 6.40% (256 participants) in the B hospital in Beijing, and 9.93% (233 participants) and 5.39% (246 participants) in the C hospital and D hospital in Guangdong. Table 1 shows the demographic characteristics of the responding healthcare workers. A few participants’ demographic information was missing (1.6–9.3%). Among the 973 participants, 326 (33.5%) were men and 621 (63.8%) were women. The number of nurses was largest reaching 362 (37.2%). Among the remaining individuals, 315 (32.4%) were doctors, 112 (11.5%) were medical technicians, and 108 (11.1%) were administrators. With respect to age group, there were 286 (29.4%) participants aged 25–30 years and only 16 (1.6%) were aged 55 years or older. Regarding education level, 411 (42.2%) participants had obtained an undergraduate degree, 174 (17.9%) participants had obtained a master’s degree, 208 (21.4%) participants had graduated from junior college, and 39 (4.0%) participants had obtained a doctorate. 394 (40.5%) respondents had a trainee title and 267 (27.4) respondents had an entry-level title. 114 (11.7%) had a mid-career title, and 108 (11.1%) were senior employees. In terms of seniority, 182 (18.7%) participants had worked less than 3 years, 226 (23.2%) participants had worked 3–5 years, and 229 (23.5%) participants had worked 6–10 years.

**Table 1:** Demographic characteristics of participating healthcare workers (*N* = 973)

Characteristic	N	%
**Sex**		
Male	326	33.5
Female	621	63.8
**Age (y)**		
< 25	70	7.2
25 ~ 30	286	29.4
31 ~ 35	243	25.0
36 ~ 40	134	13.8
41 ~ 45	95	9.8
46 ~ 50	69	7.1
51 ~ 55	44	4.5
56 ~ 60	16	1.6
**Post**		
Doctors	315	32.4
Nurses	362	37.2
administrators	108	11.1
Medical technicians	112	11.5
**Education**		
Less than junior college degree	124	12.7
Junior college	208	21.4
Undergraduate	411	42.2
Master	174	17.9
Doctor	39	4.0
**Title**		
Trainee	394	40.5
Entry-level	267	27.4
Mid-level	114	11.7
Senior	108	11.1
**Seniority (y)**		
< 3	182	18.7
3 ~ 5	226	23.2
6 ~ 10	229	23.5
11 ~ 20	172	17.7
> 20	98	10.1
**Department**		
Physician	182	18.7
Surgery	139	14.3
Obstetrics/Gynecology	105	10.8
Pediatrics	118	12.1
Chinese medicine	65	6.7
Emergency Department/ICU	51	5.2
Oncology	18	1.8
Other clinical departments	56	5.8
Medical technology	73	7.5
Administration and Logistics	22	2.3
Other	71	7.3

*ICU* Intensive care unit

### Means (SD) of PSM, challenge stress, hindrance stress, supervisor support and coworker support

As shown in Table 2, the means for the 5 PSM items were very high, but the range was considerable. The means ranged from 3.40 to 4.05. The mean for the challenge stress items was higher than that for hindrance stress items. The means for the seven supervisors support and coworker support items were relatively high. They ranged from 3.39 to 3.93.

**Table 2:** Means (SD) for Public Service Motivation (PSM), Challenge Stress (CHS), Hindrance Stress (HS), Supervisor Support (SS) and Coworker Support (CS) items

Variable	Item	Mean	SD
PSM (1–5)	PSM1. Meaningful public service is very important to me.	4.05	0.76
	PSM2. I am often reminded by daily events about how dependent we are on one another.	3.91	0.82
	PSM3. Making a difference in society means more to me than personal achievements.	3.75	0.87
	PSM4. I am prepared to make sacrifices for the good of society.	3.40	1.03
	PSM5. I am not afraid to go to bat for the rights of others even if it means I will be ridiculed.	3.46	0.95
Challenge stress (1–6)	CHS1. The number of projects and/or assignments I have	3.59	0.85
	CHS2. The amount of time I spend at work	3.62	0.84
	CHS3. The volume of work that must be accomplished in the allotted time	3.51	0.88
	CHS4. Time pressures I experience	3.57	0.85
	CHS5. The amount of responsibility I have	3.63	0.87
	CHS6. The scope of responsibility my position entails	3.51	0.86
Hindrance stress (1–5)	HS1. The degree to which politics rather than performance affects organizational decisions	2.81	1.13
	HS2. The inability to clearly understand what is expected of me on the job	2.33	1.06
	HS3. The amount of red tape I need to go through to get my job done	3.18	1.06
	HS4. The lack of job security I have	3.15	1.14
	HS5. The degree to which my career seems “stalled”	3.05	1.08
Supervisor support (1–4)	SS1. My supervisor is helpful to me in getting the job done.	3.80	0.90
	SS2. My supervisor is willing to extend himself/herself to help me perform my job.	3.69	0.92
	SS3. My supervisor takes pride in my accomplishments at work.	3.51	0.91
	SS4. My supervisor tries to make my job as interesting as possible.	3.39	0.96
Coworker support (1–3)	CS1. My coworkers listen to me when I need to talk about work-related problems.	3.81	0.77
	CS2. My coworkers help me with difficult tasks.	3.83	0.78
	CS3. My coworkers help me in crisis situations at work.	3.93	0.88

“*R*” indicates a negatively phrased and reverse scored item

### Correlations between PSM, challenge stress, hindrance stress, supervisor support and coworker support

The correlation coefficients (r) among different variables were shown in Table 3. PSM was significantly negatively correlated with challenge stress and hindrance stress (*r* = − 0.39 to − 0.45) but significantly positively correlated with coworker support and supervisor support (*r* = 0.41 to 0.53). Supervisor support and coworker support were significantly negatively correlated with challenge stress (*r* = − 0.16 to − 0.11) and hindrance stress (*r* = − 0.32 to − 0.26). Correlation between coworker support and supervisor support was significantly positive (*r* = 0.60). Similarly, coefficients showed that challenge stress was significantly positive to hindrance stress (*r* = 0.53).

**Table 3:** Intercorrelations between Public Service Motivation (PSM), Challenge Stress (CHS), Hindrance Stress (HS), Supervisor Support (SS) and Coworker Support (CS) items

Variables (Mean, SD)	Variables				
	PSM	CHS	HS	SS	CS
PSM (2.33, 1.40)	1	–	–	–	–
CHS (3.53, 0.76)	−0.39**	1	–	–	–
HS (2.82, 0.88)	−0.45**	0.53**	1	–	–
SS (3.61, 0.85)	0.53**	− 0.16**	− 0.32**	1	–
CS (3.87, 0.70)	0.41**	−0.11**	−0.26**	0.60**	1

**p < 0.01

Cohen proposed criteria for different effect value of correlation coefficients [55]. In this article, *r* = 0.60 (between coworker support and supervisor support) and *r* = 0.53(between PSM and supervisor support) can be recognized as large effect. *r* = − 0.11 (between coworker support and challenge stress) and *r* = 0.16 (between supervisor support and challenge stress) can be considered as small effect. The other r should be divided into medium effect.

### SEM

Before observing the coefficients of SEM, we first analyzed the model fit. The values for the goodness-of-fit index and comparative fit index were between 0.917 and 0.952, which means that the model fits data well.

**Fig. 2:**
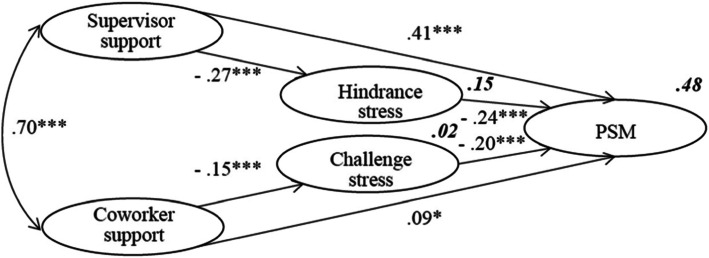
Final Model Illustrating How Supervisor Support and Coworker Support Influence Challenge Stress, Hindrance Stress and Public Service Motivation (PSM). Numbers not in bold are standardized regression coefficients and numbers in bold explain variability Chi-square = 814.315, degrees of freedom = 0.215, *p* < .001, root mean square error of approximation = 0.054, normed fit index = 0.953; comparative fit index = 0.965. *p < 0.05, ***p < 0.001

In the results of model (Fig. 2), challenge stress (*β* = − 0.20; *p* < .001) and hindrance stress (*β* = − 0.24; *p* < .001) were directly negatively associated with PSM. Supervisor support was significantly positively associated with PSM (*β* = 0.41; *p* < .001), and the path from coworker support to PSM was significant (*β* = 0.09; *p* < .05). There was a direct positive association between supervisor support and coworker support (*β* = 0.70; *p* < .001). Supervisor support was significantly negatively associated with hindrance stress (*β* = − 0.27; *p* < .001), and coworker support was significantly negatively associated with challenge stress (*β* = − 0.15; *p* < .001). All paths were significant, so that the relationship between these variables did exist. The coefficient between supervisor support and coworker support was the largest which meant that their relationship was greatest. Supervisor support and coworker support explained 15 and 2% of the variability in hindrance stress and challenge stress. Low percentages of variability are common when the outcome variable is perception, attitude, or behavior [56]. Compared with coworker support to explain challenge stress, supervisor support can better explain hindrance stress. Supervisor support, coworker support, challenge stress and hindrance stress explained 48% of the variability in PSM.

With respect to Sobel test, we noticed that there were significant indirect effects between supervisor support and PSM (Sobel *z* = 5.64; *p* < .001) and between coworker support and PSM (Sobel *z* = 8.39; *p* < .05) where hindrance stress and challenge stress played a mediating role, respectively.

The effect of coworker support on challenge stress, the effect of supervisor support on PSM, the effect of coworker support on PSM, and the effect of hindrance stress on PSM were different from the effect of the final model, which can be indicated in the subgroup analysis (Table 4). To be more precise, Coworker support did not significantly affect challenge stress among 41 years or older participants and was positively related to PSM among 41 years or older and mid/late career workers, women, and workers with more than 5 years of seniority. It meant that the relationship between coworker support and challenge stress did not exist among 41 years or older participants but exist among other groups. Interestingly, the impact of supervisor support on PSM was not significant in doctors. They would not be affected by supervisor support on their PSM. Besides, hindrance stress was negatively related to PSM except nurses. Coworker support did not affect PSM significantly in these three occupations. The invariance might be tenable across 41 years or older /30 years or younger and early/mid-late groups since the difference between the confirmatory factors analysis of our models of varying levels of measurement invariance is less than 0.01 [57].

**Table 4:** Standardized regression coefficients (*β*) with *p*-values for the components of subgroup analyses

Paths	30 years or younger (***n*** = 356)	31–40 years (***n*** = 377)	41 years or older (***n*** = 224)	Early career (***n*** = 661)	Mid/late career (***n*** = 222)	Men (***n*** = 326)	Women (***n*** = 621)	< 5 years (***n*** = 408)	> 5 years (***n*** = 499)	Doctors (***n*** = 315)	Nurses (***n*** = 362)	Others (***n*** = 220)
**SS to HS**	−0.46***	−0.46***	− 0.28***	−0.44***	− 0.34***	−0.38***	− 0.39***	−0.33***	− 0.40***	−0.45***	− 0.39***	−0.42***
**CS to CHS**	−0.21***	−0.12***	0.288^ns^	−0.20***	− 0.11*	−0.14*	− 0.16***	−0.13*	− 0.15**	−0.22**	− 0.14*	−0.14*
**SS to PSM**	0.39***	0.39***	0.33***	0.42***	0.34***	0.32***	0.48***	0.34***	0.42***	0.051^ns^	0.54***	0.50***
**CS to PSM**	0.572^ns^	0.572^ns^	0.26 **	0.294^ns^	0.15 *	0.527^ns^	0.11 *	0.719^ns^	0.14**	0.199^ns^	0.111^ns^	0.442^ns^
**CHS to PSM**	−0.29***	−0.29***	−0.17***	− 0.21***	−0.21***	− 0.22***	−0.23***	− 0.25***	−0.28***	− 0.29***	−0.23***	− 0.28***
**HS to PSM**	−0.29***	− 0.29***	−0.17***	− 0.33***	−0.20***	− 0.33***	−0.16***	− 0.24***	−0.15***	− 0.36***	0.210^ns^	− 0.25***
**CS to HS**	0.67***	0.67***	0.76***	0.62***	0.76***	0.74***	0.67***	0.73***	0.68***	0.77***	0.58***	0.73***

*ns* not statistically significant. PSM stands for Public Service Motivation, CHS stands for Challenge Stress, HS stands for Hindrance Stress, SS stands for Supervisor Support and CS stands for Coworker Support

*p < 0.05, **p < 0.01, ***p < 0.001

## Discussion

Using a sample of 973 healthcare workers, we investigated the relationship between social support, job stress and PSM in Chinese public hospitals. Overall, we found comprehensive but mixed support for our initial hypotheses. Supervisor support and coworker support significantly increased PSM among healthcare workers. Challenge stress and hindrance stress were negatively associated with PSM. Supervisor support and coworker support diminished hindrance stress and challenge stress, respectively, among Chinese healthcare workers. Challenge stress and hindrance stress significantly mediated the indirect effects of coworker support and supervisor support, respectively, on PSM. The present findings should prove useful for academic researchers and practitioners.

Our first key finding was that social support and job stress were predictors of PSM of healthcare workers, which added to the evidence from antecedent research on PSM. Social support, especially supervisor support and coworker support, has been used to explain a wide range of individual values, attitudes and behaviors, such as affective commitment [58], presenteeism [15], However, limited studies have investigated the relationship between social support and PSM. Our study provided empirical evidence of the positive effects of supervisor and coworker support on PSM. However, multi-group analysis showed that the impact of coworker support on PSM was weaker than that of supervisor support. This result is likely attributable to the fact that public hospitals are the main provider of healthcare services in China. Healthcare workers in China are exposed to high workloads and health problem but get paid less compared with their counterparts in Europe and the US [15]. An imbalance between job demand and resources limits the PSM of healthcare workers and eliminates the effect of coworker support on PSM [30]. In the light of context above, administrators should encourage and assist supervisors in their leadership functions, e.g., by urging supervisors to care about the work and life of subordinates and assist them whenever possible, identify and acknowledge the strengths and accomplishments of workers and encourage and recognize their efforts, and make workers’ jobs as interesting as possible [59]. Besides, they should also emphasize coworker support and good employee relationships. For instance, a system of group activities in public hospitals could be developed in order to enhance coworker support. Such a system could include training activities and outward-bound activities and might prevent conflicts and improve career development.

Our findings showed that supervisor support and coworker support had significant negative effects on hindrance stress and challenge stress of healthcare workers, respectively, which supported our previous hypotheses. The relationship between social support and job stress is multipath [60]. Social support and job stress are both multidimensional concepts, with the development of research, scholars are not only concerned with the overall concept but also the relationship between different dimensions which is multipath. Some previous studies have proved that supervisor support can reduce job stress [61–63], and coworker support can also play the same role [64, 65], which was consistent with our findings. Supervisor support and coworker support can be viewed as job resources that deals with job stress through reducing psychological costs and promoting personal development according to JD-R theory [66]. However, these studies regarded stress as a unidimensional conceptualization and ignored the possible positive effect of stress [67, 68]. Consequently, to complement these previous findings, stress was divided into challenge stress and hindrance stress in this study. We investigated separately the relationships between supervisor support and hindrance stress, and between coworker support and challenge stress. Our results suggested that supervisor support lessened the hindrance stress of healthcare workers, because they feel they are taken seriously by their supervisors which indicates that organizational decisions are affected by their performance rather than politics so that the work environment is fair. Besides that, supervisor will explain the job to employees to help them understand and perform job, and provide job security. All these factors consist of the antitheses of hindrance stress. In contrast, coworker support decreased challenge stress among healthcare workers, because when healthcare workers cooperate with others, they can complete their duties—such as diagnosis and treatment—faster and more effectively, which lightens their psychological burden and sense of urgency [22].

Challenge stress and hindrance stress had a significantly direct negative effect on PSM of healthcare workers. Since individuals under severe job stress are less sensitive to others, they are unwilling and impatient to help. Therefore, job stress increased aggressiveness and weakened PSM [69, 70]. Previous studies have claimed that PSM can reduce the job stress on employees in the public service sector [2]. However, Giauque et al. believe that the current research on work stressors and PSM is far from enough. The relationship explained at this stage is much simpler than the real mechanism. Therefore, more empirical articles are needed to explore this topic [71].. Nevertheless, there are currently few articles exploring the role of job stress on PSM. Our research eliminated this limitation and found the negative relationship between job stress and PSM. On the one hand, the present findings showed that challenge stress had a significant negative effect on PSM and that the effect was as strong as that of hindrance stress. This result differs from our hypothesis. Since challenge stress always has a positive effect on individuals, e.g., by encouraging workers to learn and to overcome obstacles, thus benefiting career development [14, 72], we proposed that challenge stress was positively related to PSM. This interesting finding might be due to the complex working environment for healthcare workers. To our knowledge, the workload of healthcare workers in China is heavy: many are pressured to work 10 or more hours almost every day and always with high efficiency and in a state of tension [72]. In addition, the duties of healthcare workers are taxing, such as the responsibility to improve and maintain patient health. Episodes of medical violence against healthcare workers have worsened both the psychological burden of healthcare workers and the healthcare environment [73]. In this context, challenge stress may exceed the threshold for benefit and is therefore unlikely to have positive effects on PSM.

On the other hand, hindrance stress showed a negative relationship with PSM, which prove our hypothesis. Although no studies have examined the relationship between hindrance stress and PSM, the adverse effects of job stress, especially hindrance stress, on the individual and organization have been widely discussed in many studies based on JD-R theory. These effects include burnout [74], increased presenteeism [72], and increased turnover intention [75]. Hindrance stress includes demands that are viewed by administrators as unnecessary impediments to personal growth and goal attainment, and by employees as insurmountable [14]. The present analysis of data from 973 healthcare workers employed in Chinese public hospitals provided direct evidence of the negative effect of hindrance stress on PSM. Nevertheless, administrators of public hospitals should still be mindful of the intense job stress of healthcare workers and undertake interventions targeting challenge stress and hindrance stress. For example, public hospitals should consider interventions that help relieve employee job stress, such as psychological counselling, physical exercise, recreational activities, and lectures on mental health.

Another important finding of this study is that while hindrance stress and challenge stress directly affected PSM, hindrance stress also mediated the effects of supervisor support, and challenge stress mediated the effects of coworker support, on PSM. In the subgroup analysis, the influences of social support on doctors’ PSM have been fully mediated by challenge stress and hindrance stress. This is mainly because doctors in China suffer from huge challenge stress (e.g., job load that can increase the psychological burden and prevalence of mental disorders) [76] and hindrance stress (e.g., job insecurity that cannot be solved and prevent career development) [77]. Severe stress makes individuals less sensitive and impatient to help others, which weakens PSM [69, 70]. Under such tremendous stress, doctors may first find job resources like supervisor support (e.g., supervisors reward doctors’ efforts to make them secured) [21] and coworker support (e.g., coworkers assist doctors’ job to reduce their job load) [22] to reduce stress according to JD-R theory. With less job stress, doctors are willing to help others and will demonstrate more PSM (e.g., altruistic behavior, dedication, and organizational citizenship behavior according to PSM theory [4]). Therefore, supervisor support and coworker support could significantly enhance PSM only through intervening challenge stress and hindrance stress among doctors. For nurses and other healthcare workers, the impact of coworker support on PSM was not significant, that is, the challenge stress played a completely intermediary role. This is because they generally play a supporting role in the team and need the help from other coworkers to reduce the challenge stress like job load [78]. With less job load, they can perform better and feel happy with their work to satisfy the psychological needs and increase the complementary fit between the individual and organization, which strengthens their motivational base for public service [19]. Therefore, like doctors, for nurses and other healthcare workers, coworker support can only increase PSM by reducing challenge stress. The mediating effect of the hindrance stress on nurses was not significant. This is mainly because nurses are always treated as support staffs, rather than formal members of the treatment team [79]. They are more eager to be treated as an important member of the team through the supervisor support rather than reducing role conflict or red tape of organizational politics. Then person-organization fit can be achieved, and they are willing to perform more altruistic behavior, dedication, and organizational citizenship behavior—the concrete manifestations of PSM [4, 19]. Hence supervisor support can increase PSM directly and the mediating effect of hindrance stress of nurses was not significant.

Because of the limited data on the antecedents of PSM, previous studies did not consider mediators between social support and PSM. Our results shed light on how supervisor and coworker support affect PSM through hindrance stress and challenge stress, as described in JD-R theory. Hindrance stress and challenge stress are the individual’s psychological reactions to job demands. Supervisor support and coworker support are important organizational resources provided to workers and help workers to meet their work demands, while reducing job stress and increasing PSM.

### Limitations and future research

This study is not without limitations. Firstly, the study was cross-sectional. Thus, the relationships between social support, job stress and PSM cannot be assumed to be causal and should be further examined in a cohort in a future longitudinal study. Secondly, we only recruited healthcare workers from Chinese public hospitals and excluded those from private hospitals, which limits the generalizability and robustness of our conclusions. Social support, job stress and PSM differ somewhat between public sector and private sector employees [80]. Therefore, to verify our hypotheses and models, future studies should investigate healthcare workers from private hospitals. Thirdly, our study considered only the effects of work-related dimensions of social support, which were mostly limited to job stress and PSM. Although the scale including supervisor support and coworker support dimensions could be used to adequately test social support, other dimensions of social support might be important in job stress and PSM. Future studies should examine how the support of family, friends and spouses affects job stress and PSM of healthcare workers. Fourthly, our study want to explain the potential psychological process of employees working in public sectors under the combined effect of job demands and job resources. However, JD-R theory proposed that social support and job stress not only affect employees’ motivation, but also significantly affect their burnout and job performance. On the basis of the results of the current study, future studies should integrate more psychological outcome variables in our research model. Finally, our sample comprises healthcare workers in a developing East Asian country and does not include healthcare workers from countries with other cultures and different levels of development. Future studies should examine whether the present findings are consistent across cultures and stages of economic development.

## Conclusions

In conclusion, supervisor support and coworker support are significantly negatively associated with hindrance stress and challenge stress, respectively, and positively with PSM. Challenge stress and hindrance stress are directly negatively associated with PSM, and the significant indirect effects between supervisor support and coworker support and PSM are significantly mediated by hindrance stress and challenge stress. Thus, administrators of public hospitals should focus on the relationship between social support, job stress and PSM and on interventions targeting increased supervisor and coworker support. In addition, administrators should attempt to decrease challenge stress and hindrance stress by improving work conditions and by offering more opportunities for healthcare workers to improve their work relationships and relieve stress.

## Supplementary Information

**Additional file 1.** Questionnaire. The questionnaire includes the scales of job stress, social support, public service motivation and demographic characteristics which were asked from participants.

## Data Availability

The datasets during and/or analyzed during the current study available from the corresponding author on reasonable request.

## Abbreviations

SS: Supervisor support
CS: Coworker support
HS: Hindrance stress
CHS: Challenge stress
PSM: Public service motivation
