# Supplementary material for: How does social support affect public service motivation of healthcare workers in China: the mediating effect of job stress
Source: BMC Public Health. 2021 Jun 5;21:1076. doi: 10.1186/s12889-021-11028-9 (PMC8180050; doi:10.1186/s12889-021-11028-9)
Supplement: Supplementary file 1 — Additional file 1. Questionnaire. The questionnaire includes the scales of job stress, social support, public service motivation and demographic characteristics which were asked from participants. [file 12889_2021_11028_MOESM1_ESM.docx]

**Questionnaire of healthcare workers' job stress, social support and public service motivation**

Dear colleagues,

Hello! Thank you for your participation in this survey. All the questionnaires will be filled in an anonymous way. The contents of the survey are used only for **scientific research**, and **absolute confidentiality**. If we have **your consent**, please fill in **every question** according to your actual situation. It will take you about **3-5 minutes** to complete the questionnaire. Your participation is the **biggest support** for our research. We sincerely thank you for your **understanding and cooperation**!

1. For this part, please think about your public service motivation and **tick the corresponding numbers.**

| **Declare to what extend do you agree to the following statements.** | **Totally disagree** | **Slightly disagree** | **Moderately** | **Slightly agree** | **Strongly agree** |
| --- | --- | --- | --- | --- | --- |
| 1.1 Meaningful public service is very important to me. | 1 | 2 | 3 | 4 | 5 |
| 1.2 I am often reminded by daily events about how dependent we are on one another. | 1 | 2 | 3 | 4 | 5 |
| 1.3 Making a difference in society means more to me than personal achievements. | 1 | 2 | 3 | 4 | 5 |
| 1.4 I am prepared to make sacrifices for the good of society. | 1 | 2 | 3 | 4 | 5 |
| 1.5 I am not afraid to go to bat for the rights of others even if it means I will be ridiculed. | 1 | 2 | 3 | 4 | 5 |

2. For this part, please think about your job stress and **tick the corresponding numbers.**

| **How do you think about the following statements?** | **No**  **stress** | **little**  **stress** | **Moderately** | **Much**  **stress** | **A great deal of stress** |
| --- | --- | --- | --- | --- | --- |
| 2.1 The number of projects and/or assignments I have. | 1 | 2 | 3 | 4 | 5 |
| 2.2 The amount of time I spend at work. | 1 | 2 | 3 | 4 | 5 |
| 2.3 The volume of work that must be accomplished in the allotted time. | 1 | 2 | 3 | 4 | 5 |
| 2.4 Time pressures I experience. | 1 | 2 | 3 | 4 | 5 |
| 2.5 The amount of responsibility I have. | 1 | 2 | 3 | 4 | 5 |
| 2.6 The scope of responsibility my position entails. | 1 | 2 | 3 | 4 | 5 |
| 2.7 The degree to which politics rather than performance affects organizational decisions. | 1 | 2 | 3 | 4 | 5 |
| 2.8 The inability to clearly understand what is expected of me on the job. | 1 | 2 | 3 | 4 | 5 |
| 2.9 The amount of red tape I need to go through to get my job done. | 1 | 2 | 3 | 4 | 5 |
| 2.10 The lack of job security I have. | 1 | 2 | 3 | 4 | 5 |
| 2.11 The degree to which my career seems “stalled”. | 1 | 2 | 3 | 4 | 5 |

3. Please think about your social support and **tick the corresponding numbers.**

| **Declare to what extend do you agree to the following statements.** | **Totally disagree** | **Slightly disagree** | **Moderately** | **Slightly agree** | **Strongly agree** |
| --- | --- | --- | --- | --- | --- |
| 3.1 My supervisor is helpful to me in getting the job done. | 1 | 2 | 3 | 4 | 5 |
| 3.2 My supervisor is willing to extend himself/herself to help me perform my job. | 1 | 2 | 3 | 4 | 5 |
| 3.3 My supervisor takes pride in my accomplishments at work. | 1 | 2 | 3 | 4 | 5 |
| 3.4 My supervisor tries to make my job as interesting as possible. | 1 | 2 | 3 | 4 | 5 |
| 3.5 My coworkers listen to me when I need to talk about work-related problems. | 1 | 2 | 3 | 4 | 5 |
| 3.6 My coworkers help me with difficult tasks. | 1 | 2 | 3 | 4 | 5 |
| 3.7 My coworkers help me in crisis situations at work. | 1 | 2 | 3 | 4 | 5 |

4. Please specify your information **by ticking the corresponding numbers**.

4.1 Your gender: (1) Male, (2) Female

4.2 Your age: (1) Below 25 years old (2) 25-30 (3) 31-35 (4) 36-40 (5) 41-45 (6) 46-50 (7) 51-55 (8) 56-60

4.3 You belong to: (1) Doctors (2) Nurses (3) Administrators (4) Medical technicians

4.4 Your education level: (1) Less than junior college degree (2) Junior college (3) Undergraduate (4) Master (5) Doctor

4.5 Your title: (1) Trainee (2) Entry-level (3) Mid-level (4) Senior

4.6 Your Seniority: (1) <3 (2) 3～5 (3) 6～10 (4) 11～20 (5) > 20

4.7 Your department: (1) Physician (2) Surgery (3) Obstetrics/Gynecology (4) Pediatrics (5) Chinese medicine (6) Emergency Department/ICU (7) Oncology (8) Other clinical departments (9) Medical technology (10) Administration and Logistics (11) Other
